# Jervell and Lange‐Nielsen Syndrome Related Clinical Genetics and Experimental Models

**DOI:** 10.1002/pdi3.70047

**Published:** 2026-04-08

**Authors:** Yafei Zhou, Christopher L.‐H. Huang, Ying Yang, Yanmin Zhang

**Affiliations:** ^1^ National Regional Children's Medical Center (Northwest), Key Laboratory of Precision Medicine to Pediatric Diseases of Shaanxi Province, Xi'an Key Laboratory of Children's Health and Diseases Shaanxi Institute for Pediatric Diseases Xi'an Children's Hospital Affiliated Children's Hospital of Xi'an Jiaotong University Xi'an, Shaanxi China; ^2^ Physiological Laboratory and Department of Biochemistry University of Cambridge Cambridge UK; ^3^ Department of Cardiology Xi'an Children's Hospital Affiliated Children's Hospital of Xi'an Jiaotong University Xi'an, Shaanxi China

**Keywords:** deafness, Jervell and Lange‐Nielsen syndrome, *KCNE1*, *KCNQ1*, long QT syndrome

## Abstract

Jervell and Lange‐Nielsen syndrome (JLNS) is defined by electrocardiographic QT prolongation and sensorineural hearing loss, caused by homozygous or compound heterozygous variants in *KCNQ1* and/or *KCNE1*. *KCNQ1* encodes the alpha subunit Kv7.1 of the ion channels accountable for slow delayed rectifier potassium currents (*IKs*), whereas *KCNE1* encodes the beta subunit mink, which modulates the function of *IKs*. This review comprehensively summarizes the clinical features and genetic traits of JLNS. The pathogenicity of the amino acid substitutions in Kv7.1 and mink was assessed using AlphaFold. Furthermore, we systematically evaluated recent progress in experimental models of JLNS as well as phenotypic characterization and mechanistic studies across these experimental platforms, encompassing nonexcitable cell systems, genetically engineered murine models, and patient‐specific induced pluripotent cell‐derived cardiomyocytes and inner ear hair cells.

## 1 Introduction

Jervell and Lange‐Nielsen syndrome (JLNS, OMIM 220400), inherited in an autosomal recessive manner, was initially described by Dr. Anton Jervell and Fred Lange‐Nielsen in 1957 [1, 2]. The clinical characteristics include congenital sensorineural hearing loss (SNHL), prolonged QTc intervals on electrocardiography (ECG), a higher incidence of ventricular tachyarrhythmia, specifically torsade de pointes, and a heightened likelihood of sudden cardiac death [3].

*KCNQ1* and *KCNE1* are the causal genes of JLNS. Homozygous variants in *KCNQ1* or *KCNE1* and compound heterozygous variants in either *KCNQ1* or *KCNE1* significantly broaden the genetic diversity associated with JLNS [4]. The ion channel subunits encoded by *KCNQ1* and *KCNE1* generate slow delayed rectifier potassium currents (*IKs*), which are essential for endolymph production in the inner ear and cardiomyocyte action potential (AP) [5]. The K^+^ concentration is markedly increased in the endolymph, and the influx of K^+^ into hair cells upon stimulation plays a pivotal role in transducing sound into neural signals [6].

In this review, the mutated genes and variant types associated with JLNS were systematically summarized together with their associated clinical characteristics and outcomes. This information is instrumental in elucidating the connection between JLNS’s genotype and phenotype, and the findings could facilitate genetic counseling, clinical risk stratification, and the development of treatment strategies for JLNS. The experimental models were also evaluated, such as the alterations in *IKs* currents induced by JLNS‐associated variants investigated by transfecting plasmids into nonexcitable cells [7]; mouse models effectively simulate the phenotypes of JLNS related to LQTS and hearing loss and contribute to understanding the pathogenesis of JLNS and exploring potential drug therapies [6, 8]. Human induced pluripotent stem cells (iPSCs) are used to examine the cumulative effects of gene variants, assess the therapeutic efficacy of in situ correction at variant sites, and evaluate prospects for organ transplantation based on patients' genetic backgrounds [9, 10, 11].

## 2 Clinical Characteristics

Children diagnosed with JLNS typically exhibit bradycardia beginning in the fetal stage [10] and commonly present to the cardiology department with symptoms due to prolonged QT intervals and T‐wave alternans [12]. Moreover, some cases are detected incidentally when patients present with conditions such as fever, diarrhea, or hypokalemia and undergo electrocardiographic evaluation. Additionally, patients with JLNS are referred to the otorhinolaryngology department owing to failed newborn hearing tests or clinical indicators of deafness, such as unresponsiveness to sound stimuli [13, 14]. Patients with severe JLNS show a high risk of malignant ventricular arrhythmia in the form of ventricular fibrillation and sudden cardiac death. For such severely affected patients with JLNS, the implantation of an implantable cardioverter‐defibrillator (ICD) can be considered based on the specific circumstances [12]. Meanwhile, some patients display gastrointestinal symptoms, secondary hypochromic anemia, and gross motor developmental delay [15, 16].

The efficacy of beta‐blockers and left cardiac sympathetic denervation is limited in patients with JLNS, as arrhythmia persists in 51%–85% of cases despite beta‐blocker treatment [5, 17, 18, 19]. An ICD is suggested as an intervention for patients with JLNS [12, 20]. In clinical practice, patients are typically maintained on beta‐blocker therapy (e.g., propranolol, metoprolol succinate, or carvedilol) with regular follow‐up, and left cervical sympathectomy is planned if malignant ventricular arrhythmias recur [12]. Alternatively, atrial pacing combined with beta‐blocker therapy has been used in patients with JLNS who were later upgraded to a dual‐chamber ICD without complications [17]. Half of the children with JLNS exhibit arrhythmia by 3 years of age, and 90% of patients display symptoms before they reach the age of 18 years. Additionally, activities such as exercise, including swimming, emotional states, auditory stimuli, anesthesia, and feverish conditions have been identified as potential triggers of cardiac arrhythmia in children with JLNS [5, 21].

## 3 Causal Genes

The causal genes of JLNS are the potassium ion channel complex *KCNQ1* and *KCNE1*. We conducted a comprehensive search of JLNS‐related variant sites in the Human Gene Variant Database (HGMD) (https://www.hgmd.cf.ac.uk/) and ClinVar (https://www.ncbi.nlm.nih.gov/clinvar), systematically classified the variant patterns, identified relevant clinical reports, and subsequently correlated these mutations with their corresponding functional regions from the UniProt database (https://www.uniprot.org/uniprotkb).

**FIGURE 1:**
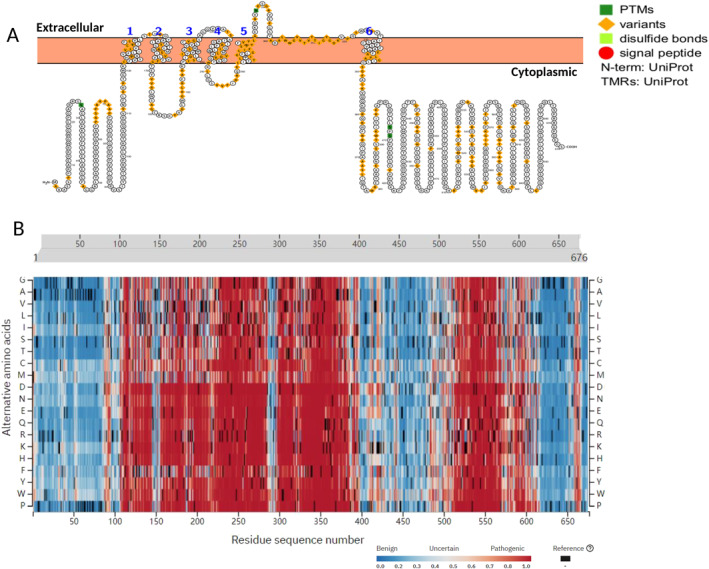
(A) Kv7.1 domain structure in Protter (http://wlab.ethz.ch/protter). Dark green marks post‐translational modification (PTM); orange marks variants registered on the website; light green marks disulfide bonds; and red marks signal peptide. (B) Prediction of the likelihood of pathogenicity of all possible amino acid substitutions of the Kv7.1 protein in AlphaFold (https://alphafold.ebi.ac.uk.). The “PDB” text of Kv7.1 was inputted into AlphaFold, and the website’s algorithm systematically substituted amino acids at each position of the protein with alternative residues to predict functional changes. The horizontal axis denotes the amino acid position, and the vertical axis indicates the corresponding amino acid name. Furthermore, the gradient from blue to red denotes an escalating severity level.

To date, a broad array of variants in *KCNQ1* have been linked to JLNS, including missense and nonsense variants, small deletions, splicing variants, small insertions, small indels, gross deletions, gross insertions, and complex rearrangements (Table 1). The analysis of the types and domain locations of *KCNQ1* variants associated with JLNS suggests that JLNS‐related variants predominantly cluster within the cytoplasmic domain, transmembrane domain, and voltage‐sensitive region in missense and nonsense mutations (Table 1, Figure 1).

**TABLE 1:** *KCNQ1* variants in Jervell and Lange‐Nielsen syndrome.

Gene	Nucleotide	Protein	Mutation type	Domain positions	Gender (female [F]/male [M])	Clinical information	Age at onset (years old)	References
*KCNQ1*	c.2T>C	p.M1	Missense	Cytoplasmic	F	QTc: 524 ms	4	Wang et al. [22]
*KCNQ1*	c.546C>A	p.S182R	Missense	Cytoplasmic	M	QTc: 446 ms, deafness	6	Wang et al. [23]
*KCNQ1*	c.557G>A	p.G186D	Missense	Cytoplasmic	M	QTc: 600 ms, deafness	2	Vyas et al. [24]
*KCNQ1*	c.604G>A	p.D202N	Missense	Helical, S3	F	QTc: 579 ms, deafness	/	Wang et al. [25]
*KCNQ1*	c.728G>A	p.R243H	Missense	Helical, voltage sensor, S4	F	QTc: 498 ms, deafness	2	Mohammad‐Panah et al. [26]
*KCNQ1*	c.775C>G	p.R259G	Missense	Cytoplasmic	F	QTc: 590 ms, deafness, syncope	2	Coto et al. [27]
*KCNQ1*	c.783G>C	p.E261D	Missense	Cytoplasmic	/	QTc: 500 ms, deafness, syncope	1.5	Tranebjaerg et al. [2]
*KCNQ1*	c.815G>A	p.G272D	Missense	Helical, S5	F	QTc: 650 ms, deafness	/	Tyson et al. [28]
*KCNQ1*	c.914G>C	p.W305S	Missense	Pore‐forming, segment H5	M	QTc: 520–560 ms, deafness	/	Neyroud et al. [29]
*KCNQ1*	c.1040T>G	p.L347R	Missense	Helical, S6	M	QTc: 470–500 ms, deafness, syncope, bradycardia	4	Matsuda et al. [30]
*KCNQ1*	c.1051T>C	p.F351L	Missense	Cytoplasmic	M	QTc: 600 ms, deafness	3.5	Vyas et al. [24]
*KCNQ1*	c.115G>T	p.E39*	Nonsense	Cytoplasmic	F	QTc: 780 ms (44 Y), deafness, syncope, epilepsy (29 Y), ICD (38 Y)	3	Nishimura et al. [31]
*KCNQ1*	c.1588C>T	p.Q530*	Nonsense	Cytoplasmic	/	/	/	Tranebjaerg et al. [2]
*KCNQ1*	c.1741A>T	p.K581*	Nonsense	Cytoplasmic	M	QTc: 661 ms, deafness, cochlear implantation, epilepsy	0.8	Qiu et al. [32]
*KCNQ1*	c.443delA	p.(Y148Lfs*89)	Small deletions	Helical, S2	M	QTc: 515 ms, deafness	2	Vyas et al. [24]
*KCNQ1*	c.451_452delCT	p.(L151Gfs*133)	Small deletions	Helical, S2	M	QTc: 520 ms, deafness	/	Chen et al. [33]
					F	QTc: 660 ms, deafness	/	
*KCNQ1*	c.585delG	p.(K196Sfs*41)	Small deletions	Cytoplasmic	F	QTc: 579 ms, deafness	/	Wang et al. [25]
*KCNQ1*	c.733_734delGG	p.(E245Rfs*39)	Small deletions	Helical, voltage sensor, S4	F	QTc: 540–560 ms, deafness, cochlear implantation	3	Amirian et al. [34]
*KCNQ1*	c.820_830del11	p.(I274Vfs*7)	Small deletions	Helical, S5	F	QTc: 528 ms, deafness, fetal bradycardia, fetal distress	2	Al‐Aama et al. [13]
*KCNQ1*	c.998_999delCT	p.(S333Cfs*129)	Small deletions	Helical, S6	/	/	/	Chung et al. [35]
*KCNQ1*	c.1008delC	p.(I337Sfs*17)	Small deletions	Helical, S6	/	/	/	Tranebjaerg et al. [2]
*KCNQ1*	c.1189delC	p.(R397Gfs*22)	Small deletions	Cytoplasmic	M	QTc: 540–560 ms, deafness	3	Wei et al. [36]
*KCNQ1*	c.1319delT	p.(V440Afs*26)	Small deletions	Cytoplasmic	F	QTc: 590 ms, deafness, syncope	2	Gao et al. [37]
			Small deletions		M	QTc: 600 ms, deafness, syncope	2	
*KCNQ1*	c.1356delG	p.(L453Wfs*13)	Small deletions	Cytoplasmic	F	QTc: 516 ms, deafness, syncope	5	Vojdani et al. [38]
*KCNQ1*	c.567dupG	p.(R190Afs*95)	Small insertions	Cytoplasmic	F	QTc: 610 ms, deafness, fetal bradycardia	0.1	Splawski et al. [39]
*KCNQ1*	c.1149dupT	p.(A384Cfs*79)	Small insertions	Not yet available	F	QTc: 524 ms	4	Wang et al. [22]
*KCNQ1*	c.743_744delGGinsTC	p.(W248F)	Small indels	Helical, voltage sensor, S4	F	QTc: 524 ms, syncope, deafness, polymorphic ventricular tachycardia, or fibrillation	4	Ohno et al. [40]; Matsuda et al. [30]
*KCNQ1*	c.1630_1635delCAGTACinsGTTGAGA	p.(Q544Vfs*108)	Small indels	Cytoplasmic	F/M	QTc: > 460 ms, deafness	/	Neyroud et al. [41]
*KCNQ1* gDNA	c.165_187del23	p.(G57Cfs*220)	Gross deletions	Cytoplasmic	/	/	/	Chae et al. [42]
*KCNQ1* cDNA	Incl. ex. 7–10	Not yet available	Gross deletions	Not yet available	M	QTc: 530 ms, deafness	1.2	Sung et al. [43]
*KCNQ1* gDNA	Insertion of 52 bp in ex. 15	Not yet available	Gross insertions	Not yet available	F	QTc: > 800 ms, deafness, syncope	6	Bersell et al. [44]
*KCNQ1* gDNA	Duplication of 86 bp c.1032_1117	Not yet available	Gross insertions	Not yet available	F	QTc: 460 ms, deafness, syncope	2	Gao et al. [37]; Kim et al. [45]
*KCNQ1*	c.853_861delins22	Not yet available	Complex rearrangements	Not yet available	F	QTc: 590 ms, deafness, syncope	0.9	Coto et al. [27]
*KCNQ1*	c.478‐2A>T	p.(E160fs + 138*)	Splicing mutations	Not yet available	/	/	/	Zhang et al. [9]
*KCNQ1*	c.477+1G>A	Not yet available	Splicing mutations	Not yet available	/	/	/	Donger et al. [46]
*KCNQ1*	c.921‐1G>A	Not yet available	Splicing mutations	Not yet available	M	QTc: 551 ms, deafness	/	Baek et al. [47]
*KCNQ1*	c.1251+1G>T	Not yet available	Splicing mutations	Not yet available	F	QTc: 528 ms, deafness, fetal bradycardia, fetal distress	2	Al‐Aama et al. [13]
*KCNQ1*	c.1686‐9T>C	Not yet available	Splicing mutations	Not yet available	M	QTc: 560 ms, deafness, fetal bradycardia	0.1	Torrado et al. [14]
*KCNQ1*	c.1686‐1G>A	Not yet available	Splicing mutations	Not yet available	/	/	/	Tyson et al. [28]
*KCNQ1*	c.1733‐1G>C	Not yet available	Splicing mutations	Not yet available	F	QTc: 515 ms, deafness	2	Vyas et al. [24]

*KCNQ1* is located on chromosome 11p15.5, and it consists of 16 exons (NM_000218) [48]. *KCNQ1* encodes potassium ion channels, specifically the alpha subunit of *IKs*. *KCNQ1* is expressed in various tissues, including cardiac tissue, the stria vascularis located within the cochlear duct [49, 50], and epithelial cells [51]. The predominant isoform of human *IKs* channels is a tetrameric protein consisting of 676 amino acids formed through the assembly of four alpha subunits. Each subunit comprises six transmembrane segments, designated S1–S6, in addition to the N‐ and C‐termini (Figure 1A). In each subunit, Segments S1–S4 form the voltage‐sensing domain, where the positively charged arginine residues on S4 serve as the main voltage sensors. Upon cell depolarization, these voltage sensors undergo an outward shift, triggering the opening of the *IKs* channel and facilitating K^+^ efflux [52]. We used AlphaFold (https://alphafold.ebi.ac.uk) to assess the pathogenicity of all possible amino acid substitutions in Kv7.1. The findings indicated that variants located near the transmembrane domain and voltage‐sensitive region exhibit heightened pathogenic potential (Figure 1B).

Studies on the current density and channel dynamics of *IKs* have demonstrated that variations in *KCNQ1* result in reduced current density due to improper assembly of *IKs* channels, leading to AP prolongation. This may contribute to the manifestation of LQT in patients [53, 54]. Zehelein et al. demonstrated that a *KCNQ1* variant in intron 1 led to partial exon 2 skipping in the mRNA of the patient’s lymphocyte. Consequently, this resulted in the generation of truncated Kv7.1 potassium channel subunits. Functional analyses performed in *Xenopus* oocytes revealed that these variant subunits exerted substantial dominant‐negative effects, markedly inhibiting *IKs* currents [55]. A homozygous variant in patients with JLNS affected transcriptional aberration; however, 10% of *KCNQ1* could escape aberrant transcription, which could effectively rescue hearing in patients but not their cardiac repolarization function [56]. This subset of patients with JLNS carrying these *KCNQ1* homozygous variants exhibited LQTS but normal hearing, which was described as recessive Romano–Ward syndrome [57]. Collectively, these data indicate that *KCNQ1* variants can produce a phenotypic spectrum ranging from isolated cardiac repolarization delay to full JLNS by either quantitatively reducing functional channel number (cumulative gene effect) or generating dominant‐negative subunits that actively impair wild‐type *IKs*, with the final outcome determined by the degree of residual current in both heart and inner ear.

**FIGURE 2:**
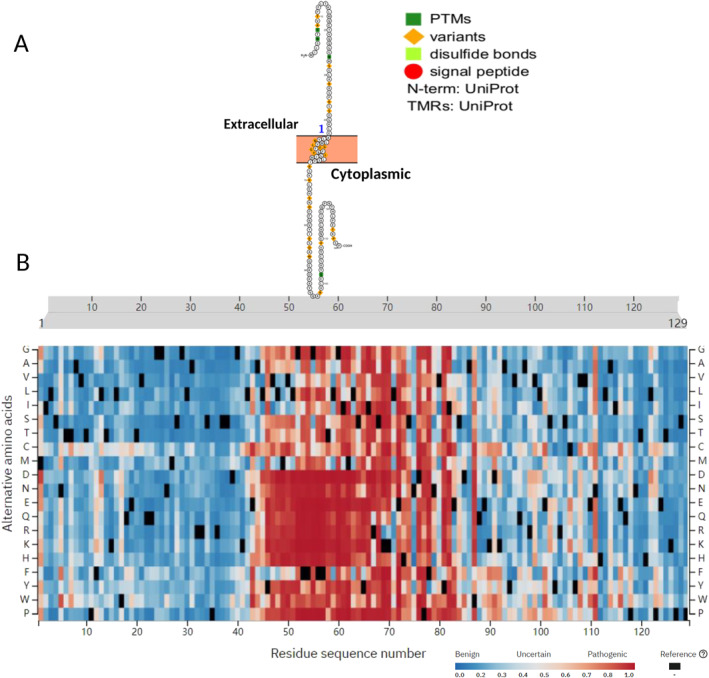
(A) Mink domain structure in Protter (http://wlab.ethz.ch/protter). Dark green marks post‐translational modification (PTM); orange marks variants registered on the website; light green marks disulfide bonds; and red marks signal peptide. (B) Prediction of the likelihood of pathogenicity of all possible amino acid substitutions of mink protein in AlphaFold (https://alphafold.ebi.ac.uk.). The “PDB” text of mink was inputted into AlphaFold, and the website’s algorithm systematically substituted amino acids at each position of the protein with alternative residues to predict functional changes. The horizontal axis denotes the amino acid position, whereas the vertical axis indicates the corresponding amino acid name. Furthermore, the gradient from blue to red denotes an escalating severity level.

*KCNE1*, located on chromosome 21q22.1‐22.2, consists of a single coding exon (NM_000219) [48]. The predominant isoform of the human channel is a tetrameric protein consisting of 129 amino acids, and *KCNE1* specifically encodes the beta subunit (mink) of *IKs* (Figure 2A). It serves as a critical regulator in the heart rate‐dependent adaptation of cardiac AP duration [58]. To date, *KCNE1* variants can be divided into seven types, including missense variants, nonsense variants, regulatory variants, small deletions, small insertions, small indels, and gross deletions. Among them, the types of JLNS‐related variants are missense variants, nonsense variants, small deletions, and small indels (Table 2). Using AlphaFold to assess the likelihood of pathogenicity of all possible amino acid substitutions in mink, we found that variants located near the transmembrane domain and voltage‐sensitive region exhibit heightened pathogenic potential (Figure 2B).

**TABLE 2:** *KCNE1* variants in Jervell and Lange‐Nielsen syndrome.

Gene	Nucleotide	Protein	Mutation type	Domain positions	Gender (female [F]/male [M])	Clinical information	Age at onset (years old)	References
*KCNE1*	c.20C>T	p.T7I	Missense	Cytoplasmic	F	QTc: 470 and 520 ms, deafness	/	Neyroud et al. [29]; Matsuda et al. [30]
			Missense		M	QTc: 480 ms, deafness	/	
*KCNE1*	c.226G>A	p.D76N	Missense	Cytoplasmic	F	QTc: 470 and 520 ms, deafness	/	Neyroud et al. [29]
			Missense		M	QTc: 480 ms, deafness	/	
*KCNE1*	c.48delG	p.(W17Gfs*47)		Helical	/	/	/	Matsuda et al. [30]
*KCNE1*	c.172_177delACCCTGinsCCCCCT	p.(Y58_L59delinsPP)	Small indels	Helical	F/M	QTc: > 460 ms, deafness	/	Ohno et al. [40]; Matsuda et al. [30]

## 4 JLNS Genotype and Phenotype Correlation

Approximately 90% of cases of JLNS are associated with *KCNQ1* variants, and the remaining cases are attributable to *KCNE1* variants [14]. Children carrying *KCNQ1* variants exhibit an approximately sixfold higher risk of ventricular arrhythmia than those with *KCNE1* variants. Conversely, heterozygous JLNS‐related variants are linked to a milder phenotype [12, 38, 59]. From an electrophysiological perspective, these findings might be related to the fact that *KCNE1* is the auxiliary regulatory subunit of *IKs* [58].

Furthermore, we analyzed the clinical data of patients with JLNS and various variant types. Of the 39 patients traced in our review, nine and five had syncope and bradycardia, respectively, in the fetal or neonatal period (Tables 1 and 2). This aligns with our previously reported findings in a child diagnosed with JLNS, in which bradycardia developed in the fetus at 23 weeks of gestation. By monitoring the fetal heart rate during perinatal examinations, bradycardia was found to persist until birth (approximately 110 bpm). After birth, the boy's QTc interval on ECG was prolonged to more than 490 ms, and he failed the hearing test. During follow‐up at 2 months of age, his ECG revealed QTc prolongation, and the hearing test revealed SNHL. The diagnosis of JLNS was confirmed by genetic testing, and the infant was immediately treated with a beta‐blocker [10]. Thus, we recommend close monitoring of the fetal heart rate during perinatal examinations to effectively avoid severe arrhythmic events. Concerning clinical cases of documented JLNS, two patients underwent cochlear implantation, which precipitated significant arrhythmic events [32, 34]. Suzuki et al. demonstrated that isoflurane inhibits *IKs* channel function, leading to a prolonged AP duration (APD) [60]. Given that the QTc interval reflects APD, it can be inferred that patients with JLNS might exhibit heightened sensitivity to anesthesia, potentially resulting in severe arrhythmic events. Consequently, stringent control over both the dosage and duration of anesthesia is recommended for patients with JLNS.

Because of incomplete clinical information, we were unable to establish a definitive relationship between the JLNS phenotype and mutation type. Thus, a substantial sample size is required to make meaningful conclusions regarding the natural progression of JLNS, genotype–phenotype correlations, risk factors for severe cardiac events, and therapeutic responses.

## 5 Experimental Models of JLNS

Beta‐blockers represent the standard of care for treating *KCNQ1* variant‐associated arrhythmia, and they have been demonstrated to minimize recurrent syncope and mortality rates in patients with LQTS Type 1 (LQT1). However, these treatments only reduce sympathetic activity in the heart without correcting the loss of function of K^+^ channels. To date, genotype–phenotype management and treatment for patients with *KCNQ1/KCNE1* variants remain unachieved [61, 62].

### 5.1 Nonexcitable Cell Lines of JLNS

Studies have utilized nonexcitable cell lines and heterologous cellular systems to explore the distinct properties of individual ion channels in *KCNQ1* and *KCNE1* variants, such as CHOs, *Xenopus* oocytes, and HEK293 human cells [7, 53, 54, 55]. Huang et al. transfected constructs carrying eight mutation sites associated with JLNS into *Xenopus* oocyte cells, demonstrating that these *KCNQ1* missense variants exert a dominant‐negative effect while retaining an intact C terminus. Specifically, the Kv7.1 variant protein exerts its dominant‐negative influence via coassembly with the WT protein, such a*s* Kv7.1 (p.E261H and p.R243H) [7]. The coassembly of frameshift mutants with a truncated C terminus and the WT protein is unlikely, thus precluding any potential dominant‐negative effect (for example, Kv7.1 [p.Y148Lfs*89]) [24]. The Kv7.1 variant proteins did not exhibit a strong dominant‐negative effect at a 1:1 injection ratio, but they did at a 1:3 ratio [7]. These findings related to JLNS indicate that the seriousness of the JLNS phenotype is influenced by the type of variant and the expression of the mutated protein. The heterologous expression system can be employed to rapidly investigate the effects of JLNS mutated genes and loci on the single‐channel current of *IKs*. Simultaneously, variants expressed in heterologous systems can be used to study variant gene dosages and dominant‐negative effects by changing the concentrations of transfected mutant plasmids [8, 55].

Consequently, when recapturing the JLNS phenotype using nonexcitable cell lines, it is essential to consider whether the concentration of the transfected mutant plasmid aligns with the expression of the mutant protein observed in patients. Nevertheless, because the expressed ion channel is solitary, it cannot restore the AP of the cell and the phenotype of LQT [7, 53]. Meanwhile, Verkerk and Wilders reported that in the human sinoatrial node cell model, under vagal tone, *IKs* current reached its minimum value, resulting in the longest cycle duration; conversely, under β‐adrenergic stimulation, *IKs* current reached its maximum value, leading to the shortest cycle duration and being significantly upregulated by cAMP [54]. However, the nonexcitable cell model lacks a comprehensive signaling network involving Ca^2+^–cAMP–PKA and Ca^2+^/calmodulin‐dependent protein kinase II‐mediated mechanisms.

### 5.2 Mouse Models of JLNS

Mouse models have been shown to be useful in elucidating the phenotypes of LQTS and SNHL, permitting exploration of the pathogenic mechanisms. Mice exhibit a similar overall heart structure as humans and possess sufficient tissue quality to accurately reproduce the polymorphic arrhythmias observed in the clinic [63]. Casimiro et al. revealed that *Kcnq1* ^−/−^ mice are deaf, and they display a characteristic shaker phenotype. A marked reduction in endolymph volume led to significant morphological abnormalities in the inner ear structures of *Kcnq1* ^−/−^ mice, as shown by histological examination. ECG in *Kcnq1* ^−/−^ mice revealed abnormal T waves and prolonged QT intervals [63]. Rivas et al. detected deafness and circling behavior in *Kcnq1* ^−/−^ mice. Notable atrophy of the stria vascularis was observed, alongside a reduction in the size of endolymphatic compartments and fusion of adjacent membranes. The organ of Corti was completely degenerated concomitantly with spiral ganglion degeneration [64].

The timing of mouse hair cell degeneration was found to depend on the specific inner ear structure that houses these cells. For example, the organ of Corti, which contains both inner and outer hair cells, displayed signs of degeneration around postnatal day 3, whereas Type I and II hair cells in the cristae started to degenerate around postnatal day 10 [8]. A significant reduction in the size of the scala media of *Kcnq1* ^−/−^ mice was onserved at 3–6 months, with pronounced degeneration occurring in the basal turn [64]. These findings indicate that early detection and intervention could significantly enhance the preservation of auditory function. Furthermore, clinical evidence demonstrates that children who receive cochlear implants at an earlier age are more likely to develop robust language systems and achieve superior outcomes [65, 66]. Because of genetic differences between humans and mice, we cannot yet assess the condition of patients with JLNS based on the development and timing of inner ear hair cell damage in mice. A mouse model with a genetic background closely resembling that of patients with JLNS was utilized to investigate the temporal patterns of functional degeneration in inner ear hair cells, thereby enhancing our understanding of the pathogenesis and potential therapeutic strategies for deafness in children with JLNS [63].

### 5.3 iPSC Models of JLNS

#### 5.3.1 Establishment of iPSC Models

In vitro reprogramming techniques enable the generation of patient‐specific iPSCs, which can be differentiated into cardiomyocytes (iPS‐CMs) and inner ear hair cells (iPS‐IHCs) to model JLNS pathology [4]. Zhou et al. established iPSC lines from patients with JLNS and their parents using immunofluorescence and flow cytometry for marker validation, providing a platform to study disease mechanisms [11, 67, 68]. Zhang et al. further engineered iPSC models with *KCNQ1* missense and splice variants, recapitulating two major JLNS‐causing defects. These iPS‐CMs exhibited prolonged APD and dose‐dependent reduction of *IKs* channels at the membrane, directly linking genotype to functional deficits [9].

#### 5.3.2 Genotype–Phenotype Relationships

Clinical observations in JLNS families reveal that homozygous *KCNQ1* variants cause LQTS and SNHL, whereas heterozygous carriers show asymptomatic or LQTS‐only phenotypes [28]. This observation suggests the need for further investigation to explore potential compensatory effects related to gene mutation dosage. JLNS iPS‐CMs differentiated from cells carrying the Kv7.1 (p.[E160fs + 138*]) homozygous mutation exhibited a significantly reduced APD. Moreover, in Kv7.1 (p.(E160fs + 138*)) heterozygous iPS‐CMs, exon 3 *KCNQ1* mRNA underwent nonsense‐mediated decay, whereas WT‐*KCNQ1* mRNA levels were elevated beyond the expected 50% level [9]. This experiment demonstrated the cumulative effect of gene mutations on clinical symptoms. Consequently, our analysis of the relationship between the genotype and phenotype of variants presented in Tables 1 and 2 indicates that the severity of the phenotype is influenced by the type and location of the mutation, and severity might be correlated with the expression of *KCNQ1/KCNE1* variants in patients. Nevertheless, this hypothesis requires further validation through additional research investigating the expression of the mutant proteins.

#### 5.3.3 Drug Sensitivity and Therapeutic Strategies in iPS‐CMs

JLNS iPS‐CMs display heightened sensitivity to adrenergic and proarrhythmic stimuli [9], consistent with clinical reports linking emotional stress or exercise to arrhythmias (e.g., syncope [31], nonsustained tachycardia [27], and T‐wave alternans [32]). Patients with LQT1 might display positive responses to beta‐blocker treatment, but this approach yields limited results in most patients with JLNS. Another option could be the use of drugs that activate *Ikr* and compensate for the impaired function of *KCNQ1* [18, 69]. The heightened susceptibility of JLNS iPS‐CMs to drug‐induced arrhythmia can be counteracted by treatment with an *Ikr* activator, highlighting the critical role of JLNS iPS‐CMs in pharmacological evaluations of drug safety and elevated‐risk drug assessment. The aforementioned studies better recapitulated the findings in humans by employing iPS‐CMs carrying the genomic information of patients with JLNS to simulate the *IKs* channel dynamics and AP phenotype of JLNS cardiomyocytes and conduct drug testing. Such approaches are advantageous for the future development of JLNS‐related 3D organoids, potentially reducing immune rejection and providing guidance for personalized medicine.

#### 5.3.4 Inner Ear Hair Cell Modeling

Mechanosensitive hair cells located within the sensory organs of the inner ear play pivotal roles in auditory and balance capabilities. In mammals, the inability of these damaged hair cells to regenerate is one of the primary factors contributing to the difficulty in treating hearing impairment in patients with JLNS [70]. Although the cardiac characteristics of JLNS were documented in several extensive retrospective and prospective studies, information regarding the inner ear phenotype in JLNS remains scarce [16, 19]. Winbo et al. introduced new clinical findings from 15 patients with JLNS, highlighting vestibular dysfunction as a key aspect of JLNS [15]. Saeki et al. demonstrated that iPSCs can be directed to differentiate into iPS‐IHCs under the combined action of fibroblast growth factor, bone morphogenetic protein, retinoic acid, and WNT signaling [70, 71]. This approach permits the replication of JLNS clinical genotypes and investigation of their underlying mechanisms. iPS‐IHCs are responsive to mechanical stimuli, exhibiting similar transduction currents and adaptation patterns as those observed in immature hair cells [70]. Although JLNS‐specific iPS‐IHC models are lacking, existing protocols enable the replication of JLNS genotypes and mechanotransduction studies. Successful differentiation would permit the in vitro simulation of JLNS variant effects (e.g., mechanical stress responses) and comparative analyses of pathogenic pathways in iPS‐IHCs versus iPS‐CMs.

## 6 Summary

This review synthesizes clinical and genetic insights, highlighting homozygous or compound heterozygous variants in *KCNQ1* or *KCNE1* as the molecular basis of JLNS. The genotypes of JLNS do not exhibit a straightforward cause‐and‐effect relationship with the severity of its clinical phenotypes in some cases. Integrated experimental platforms advance mechanistic understanding and therapeutic development, which are beneficial for JLNS management.

## 7 Limitations

As a multisystemic ion channel disorder, JLNS needs more attention during prepregnancy, pregnancy‐related, and newborn hearing screening for early diagnosis and treatment. Currently, research is limited by the lack of a global, multiethnic epidemiological database for JLNS. In the future, with such a database and integration, we hope to explore the links between genetic mutations and cardiac/auditory phenotypes in diverse JLNS populations [72]. Additionally, comparing JLNS‐related genes with other hearing loss genes (e.g., *ATB7*, *SCL26A4*, and *GJB2*) could offer new insights for clinical practice [73, 74]. However, these objectives are currently constrained by data shortages.

## Author Contributions

Yafei Zhou wrote the original manuscript. Ying Yang, Yanmin Zhang and Christopher L.‐H. Huang reviewed and edited the manuscript. All authors contributed to the article and approved the submitted version.

## Funding

This work was supported in part by the Natural Science Foundation of Shaanxi Province (Grant No. 2025JC‐YBMS‐876); the Institutional Scientific Research Projects of Xi’an Children’s Hospital (Grant No. 2024D01); the Innovative Research Group Project of the National Natural Science Foundation of China (Grant Nos. 82211530115 and 82200399); the Shaanxi Provincial International Science and Technology Cooperation Program Key Project (Grant No. 2024GH‐ZDXM‐38); and the Xi'an Science and Technology Plan Project (Grant No. 24YXYJ0027).

## Conflicts of Interest

The authors declare no conflicts of interest.

## Data Availability

The data that support the findings of this study are available upon request from the corresponding author. The data are not publicly available due to privacy or ethical restrictions.
